# Emotion Regulation as the Foundation of Political Attitudes: Does Reappraisal Decrease Support for Conservative Policies?

**DOI:** 10.1371/journal.pone.0083143

**Published:** 2013-12-18

**Authors:** Jooa Julia Lee, Yunkyu Sohn, James H. Fowler

**Affiliations:** 1 Harvard Kennedy School, Harvard University, Cambridge, Massachusetts, United States of America; 2 Department of Political Science, University of California San Diego, La Jolla, California, United States of America; 3 Division of Medical Genetics, University of California San Diego, La Jolla, California, United States of America; Center for BrainHealth, University of Texas at Dallas, United States of America

## Abstract

Cognitive scientists, behavior geneticists, and political scientists have identified several ways in which emotions influence political attitudes, and psychologists have shown that emotion regulation can have an important causal effect on physiology, cognition, and subjective experience. However, no work to date explores the possibility that emotion regulation may shape political ideology and attitudes toward policies. Here, we conduct four studies that investigate the role of a particular emotion regulation strategy – reappraisal in particular. Two observational studies show that individual differences in emotion regulation styles predict variation in political orientations and support for conservative policies. In the third study, we experimentally induce disgust as the target emotion to be regulated and show that use of reappraisal reduces the experience of disgust, thereby decreasing moral concerns associated with conservatism. In the final experimental study, we show that use of reappraisal successfully attenuates the relationship between trait-level disgust sensitivity and support for conservative policies. Our findings provide the first evidence of a critical link between emotion regulation and political attitudes.

## Introduction

A large body of research suggests that political conservatives are more likely than political liberals to express and experience negative emotions like fear and anger [1]–[5]. Recent neurophysiological studies suggest that liberals and conservatives may differ in the ways in which they process the information that induces affective states. In particular, political liberalism is associated with stronger brain activity in the dorsal region of the anterior cingulate cortex (dACC) [6], an area that detects and regulates the competition between cognitive and emotional processes [7], [8]. Similarly, Democrats are more likely than Republicans in a risky decision-making task to show activity in the left insula, a region associated with emotional self-regulation [9]. In contrast, Republicans show more activity than Democrats in the right amygdala, a part of the brain that plays an important role in emotional reactions [9]. Taken together, the accumulating empirical evidence on neurocognitive mechanisms suggests that differences between liberals and conservatives may stem from the way they process and regulate negative emotions.

Importantly, conservatives are also more likely than liberals to experience and express disgust [10]–[11]. Research has found that liberals base their moral judgments on harm avoidance and a desire for fairness, while conservatives center their judgments around three additional concerns, such as purity, in-group loyalty, and authority [12], [13]. The finding that conservatives are more likely to be concerned with purity suggests that disgust may be one critical emotion distinguishing them from liberals. In fact, experimentally manipulated disgust that is unrelated to one's moral and political judgments has been shown to increase punitive moral judgments related to purity [14]–[16] and negative feelings against out-group members, such as gay men [17], [18]. Further, individuals who are more sensitive to disgust at the trait-level are more likely to self-identify as politically conservative and to vote for the conservative party [19]. Similarly, heightened involuntary physiological arousal to disgusting images has been found to increase the likelihood that individuals support conservative policies such as gay marriage and abortion and identify as conservative [11], [20]. While these studies offer a link between support for conservative policies, political conservatism and emotion, they imply a rather deterministic process in which an individual's political attitudes are a passive reflection of one's innate emotional and biological makeup.

Here, we test the hypothesis that individuals play a proactive role in their experience of the world, and that this direct experience influences their political beliefs. We explore the possibility that there may exist fundamental differences between liberals and conservatives not only in the way that they *experience emotions*, but also in the way that they *regulate emotions*. Although some of these differences may be long-lasting or innate, focusing on the regulation of emotion rather than the experience of emotion brings up the possibility that individuals may be able to take control of their affective state, and thus may be able to influence the effect of their emotions on political orientation.

Not all emotion regulation strategies, however, are the same. Two important strategies, *reappraisal* and *suppression*, are known to have different consequences for physiology, cognition, and subjective experience [21]. More specifically, reappraisal can be employed earlier than suppression, altering one's thoughts about a target event *before* any emotion occurs. On the other hand, suppression involves concealing one's feelings *after* emotion occurs. Due to this temporal difference, reappraisal has been found to be more effective than suppression in making individuals feel less negative after the event [22], [23] and leading to less sympathetic nervous system arousal [23], [24]. In particular, the use of reappraisal has been shown to decrease the influence of emotion-driven intuitions, and in turn encourage deliberative moral judgments [25]. Thus, we reason that political attitudes are shaped by the way one deals with emotions, by either dispositional or situational use of reappraisal.

Individuals who use reappraisal more frequently will be more successful at regulating the negative emotions that have been found to increase political conservatism than those who employ reappraisal less frequently. We first used a correlational design to examine the relationship between self-reported emotion-regulation styles (frequency with which individuals use reappraisal, in particular) at the trait-level, and individual differences in political attitudes. We view that political ideology is more likely to be dispositional while policy support is more likely to be context-dependent [26]. Thus, at the trait-level, we predicted that frequent reappraisal, but not frequent suppression, is associated with reduced support for conservative policies and a decrease in general identification with political conservatism.

Given the strong relationship between disgust and political attitudes [11], [18], [19], we expected that situational reappraisal targeted at reducing the experience of disgust attenuates the tendency to derive moral and political judgments from disgust. We hypothesized that the situational use of reappraisal is less affected by disgust inducing stimuli, and thus less likely to influence moral intuitions based on purity-related concerns, which have been known to increase political conservatism [14]–[16]. We further hypothesized that situational reappraisal moderates the effect of innate disgust sensitivity on support for conservative policies by reducing the occurrence of purity-based moral judgments. We measured both the subjective and the physiological experience of disgust to show that the use of reappraisal (but not suppression) can reduce disgust at the individual-level, and that this reappraisal can carry over to affect moral and political judgments.

The Institutional Review Board at Harvard University approved all of our studies. Participants gave written informed consent prior to the study.

## Experiment 1

In Experiment 1a, we examined the association between frequent use of reappraisal and support for conservative policies. In Experiment 1b, we further examined the possibility that frequent use of reappraisal predicts self-identified political conservatism.

### Methods

#### Participants and Procedure

For Experiment 1a we recruited 120 individuals (*M_age_* = 35.21, *SD_age_* = 12.37; 50% male) from Amazon Mechanical Turk (www.mturk.com). Participants completed a 10-minute online survey, assessing their emotion-regulation styles, transient mood, and support for 32 different policies ranging from foreign immigration to gay marriage to abortion (See Text S1). All participants were U.S. residents, and received $0.30 for their participation.

In Experiment 1b, we recruited 199 adults (*M_age_* = 25, *SD_age_* = 4.03; 53% male) from the Boston/Cambridge area to participate in a 15-minute survey, which was part of a series of unrelated studies. The study took approximately one hour to complete and participants were compensated with $20. To minimize potential demand characteristics that may result from participants' expectations about the relationship between emotion, emotion regulation, and political ideology, we gathered data at separate stages, making it difficult for participants to guess the research hypothesis. First, we assessed political orientation from our study pool's general demographic survey. Once participants signed up specifically for our study, they completed a questionnaire assessing individual differences in state emotions and emotion regulation styles. We then matched the responses from the demographic survey on political orientation to our main questionnaire on emotion regulation style.

We also included a series of questions about moral decisions at the end of the questionnaire. However, we chose not to report the analyses in this paper, as they are not pertinent to our core hypotheses and do not influence our results.

#### Measures

In both Experiment 1a and 1b, we assessed participants' emotion-regulation style by asking them to indicate the extent to which they agree with 10 items on the Emotion Regulation Questionnaire (ERQ) [23]. The items were rated from 1 (*strongly disagree*) to 7 (*strongly agree*). ERQ measures the frequency with which respondents use two emotion-regulation styles: suppression (e.g., “I control my emotions by not expressing them”; *α* = 0.83 in Experiment 1a, *α* = 0.76 in Experiment 1b) and reappraisal (e.g., “I control my emotions by changing the way I think about the situation I'm in”; *α* = 0.90 in Experiment 1a, *α* = 0.84 in Experiment 1b).

In Experiment 1a, participants were asked to indicate their support for 32 political issues using a 3-point scale, ranging from 1 (*yes*), to 2 (*unsure*), to 3 (*no*). This measure has been used to gauge support for conservative policies [27]. The summary variable was operationalized to indicate the extent to which one supports conservative policies (*α* = 0.86). We used principle components analysis to find the one-factor solution that retained all items and calculated regression-factor scores for each participant (See Table S1 in Text S1 for the list of policies as well as factor analysis results). For Experiment 1b, political orientation was measured on a scale from 1 (*very liberal*) to 7 (*very conservative*). We paired self-reported political orientations from the pre-screening survey with data from our demographic survey using the unique participant ID.

Lastly, in both Experiment 1a and 1b, we asked participants to indicate their sex, age, level of education (1 =  High school, 2 =  Some College, 3 =  Associate's Degree, 4 =  Bachelor's Degree, 5 =  Post Grad, 6 =  Master's Degree, 7 =  Doctoral Degree, 8 =  None of the above) and monthly household income (1 =  None, 2 =  Under $60, 3 = $60–499, 4 = $500–999, 5 = $1,000–1,999, 6 = $2,000–2,999, 7 = $3,000–3,999, 8 = $4,000–4,999, 9 = $5,000–7,499, 10 = $7,500–9,999, 11 =  Over $10,000, 12 =  Don't know/Prefer not to answer). Also, we assessed participants' positive and negative affect to account for transient differences in state emotions. We used the 12-item form of the Positive and Negative Affect Scale (PANAS) [28]. Participants indicated the extent to which they felt a specific emotion “right now” using a 5-point scale from 1 (*very slightly or not at all*) to 5 (*extremely*).

### Results

#### Experiment 1a

**Figure 1:**
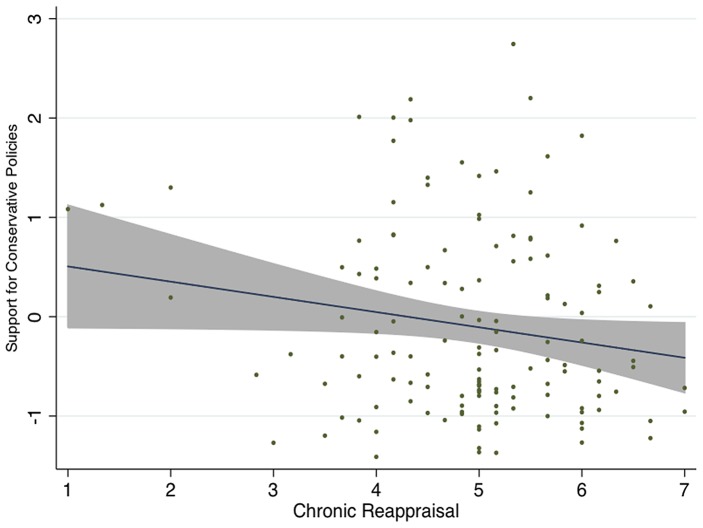
Chronic reappraisal is negatively associated with self-reported political conservatism, Experiment 1 (shaded area  = 95% CI).

Consistent with our hypothesis that one specific type of emotion regulation (reappraisal) would be uniquely related to one's support for conservative policies, we found in Experiment 1a that frequent reappraisal is negatively associated with support for conservative policies (*r* = –0.22, *p* = 0.01; See Fig. 1). On the other hand, the relationship between suppression and support for conservative policies is weak and not significant (*r* = –0.03, *p* = 0.71; See Table S2 in Text S1). In the Table S3 in Text S1 we present results from several multiple regression analyses to demonstrate that the relationship between emotion regulation styles and support for conservative policies is *specific* to reappraisal but not to suppression. Lastly, the negative relationship between reappraisal and support for conservative policies was robust to controls for key demographics, such as age, education, income, and sex, and transient positive and negative mood.

#### Experiment 1b

**Figure 2:**
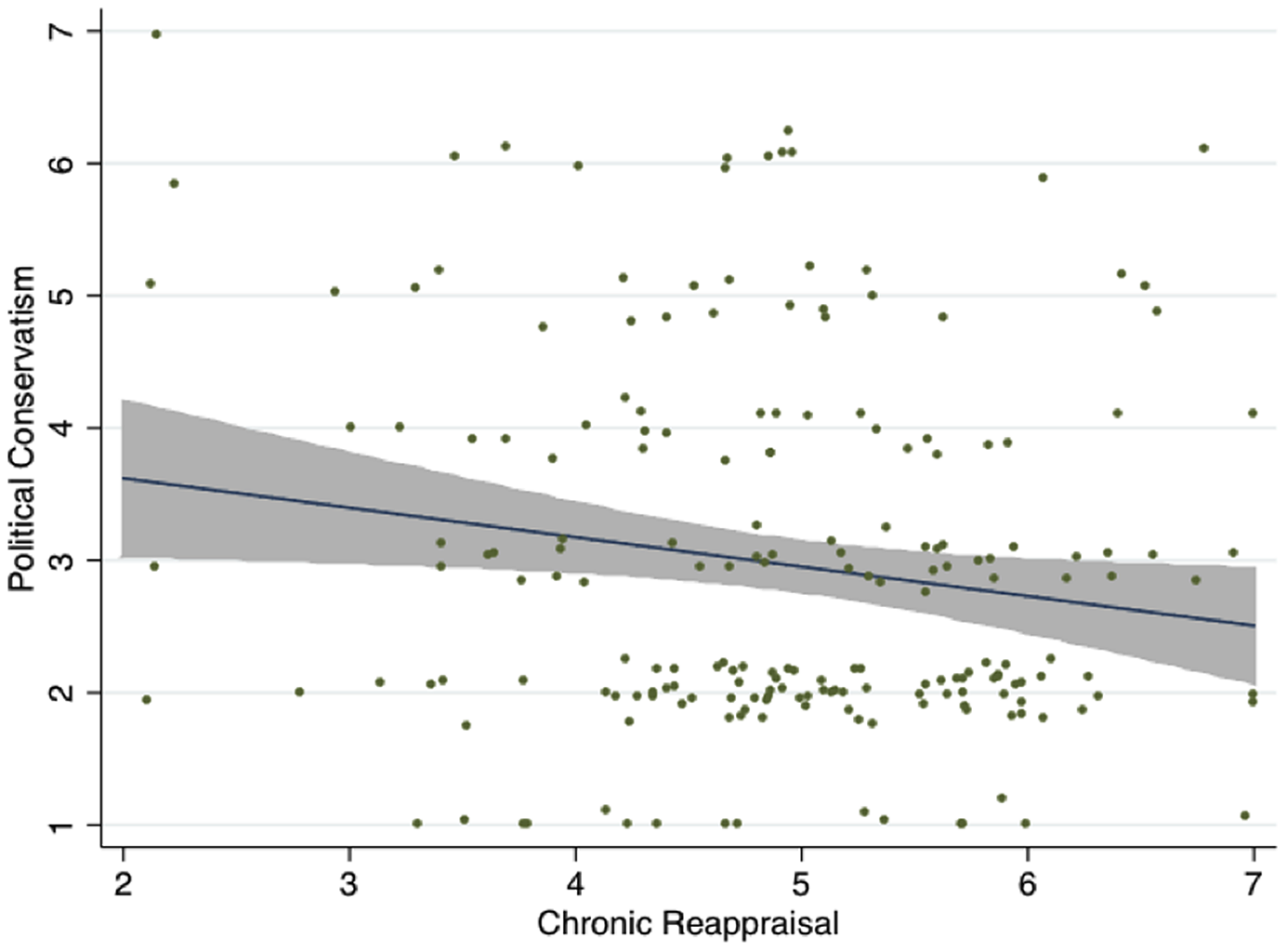
Chronic reappraisal is negatively associated with support for conservative policies, Experiment 2 (shaded area  = 95% CI).

Fig. 2 shows that participants in Experiment 1b who habitually used reappraisal were significantly less likely to self-identify as conservative (*r* = –0.16, *p* = 0.02; See Table S4 in Text S1) than those who used suppression (*r* = 0.15, *p* = 0.04). After testing for both reappraisal and suppression, only reappraisal continued to be a significant predictor of conservatism (B = –0.22, *p* = 0.04). Using the same regression models in Experiment 1a, we accounted for potential effects of key demographics and transient mood. Even after taking these controls into account, the direction, magnitude, and significance of the relationship between reappraisal and conservatism did not change (See Table S5 in Text S1).

Taken together, these observational studies suggest that reappraisal is negatively associated with both support for conservative policies and self-identified political conservatism.

## Experiment 2

In Experiment 2, we designed an experiment to ascertain whether this relationship is causal and to test whether reappraisal can be used to regulate specific emotions, such as disgust. Instead of asking participants to self-report how *often* they engage in emotion regulation, we randomly assigned them to three different treatments [29]. In the first treatment, we asked participants to employ reappraisal as an emotion regulation strategy. In the second treatment, we asked them to employ suppression. In the third treatment (the control group) we did not prompt them to use any emotion regulation strategy.

### Methods

#### Participants and Procedure

We recruited 139 individuals (*M_age_* = 38.8, *SD_age_* = 13.6; 56% male) from Amazon Mechanical Turk to participate in a 20-minute online study. Participants were randomly assigned to one of three conditions: reappraisal, suppression, and control. This manipulation allowed us to assess the causal impact of emotional regulation strategies on disgust. Specifically, we were interested to see whether emotion regulation strategies can influence experience of disgust and consequently affect concerns for purity.

The instructions for the reappraisal condition appeared as follows:

*As you view the images, please try to adopt a detached and unemotional attitude. Or, you could think about the positive aspect of what you are seeing. Please try to think about what you are seeing objectively, watch all images carefully, but please try to think about what you are seeing in such a way that you feel less negative emotion.*

The instructions for the suppression condition appeared as follows:

*As you view the images, if you have any feelings, please try your best not to let those feelings show. Watch all images carefully, but try to behave so that someone watching you would not know that you are feeling anything at all.*

In the control condition, participants were asked to carefully observe a series of images.

After reading the instructions, participants in all three conditions were presented with seven photos of disgusting stimuli, such as a cockroach or a dirty toilet, taken from the International Affective Picture System (IAPS) [30]. The images appeared for 7 seconds, followed by 3-second rest period. Previous studies have used these images to induce disgust reliably [17], [31]. None of the images had any relevance to moral judgment or political attitudes.

Next, participants were asked to answer questions regarding the factors that they take into account when making moral decisions, transient mood, and standard demographic questions. As a manipulation check, we asked participants to recall their instructions and choose the one strategy they actually employed. Out of the 139 participants, 2 participants in the reappraisal condition and 15 participants in the suppression condition reported that they used the incorrect emotion regulation strategy. Therefore, we excluded these 17 participants from further analysis, as they did not understand the instructions or did not follow the instructions correctly [32]. Thus, a total of 122 participants were included in the analysis (43 reappraisers, 32 suppressors, and 47 controls).

#### Measures

We used the shortened Moral Foundations Questionnaire (16-item MFQ Part I) [33] to assesse the *relevance* of various principles on moral decision-making. This scale yielded scores on five distinct foundations of morality: harm (*α* = 0.82), fairness (*α* = 0.83), loyalty (*α* = 0.80), authority (*α* = 0.70), and purity (*α* = 0.67).

Also, participants were asked to indicate the extent to which they felt various emotions at the moment. This allowed us to test whether disgust stimuli influenced self-reported negative feelings, including disgust. Using the same PANAS measure as in Experiment 1 [28], we combined three related items (disgusted, repulsed, nauseated) and observed the following summary variables: post-stimuli negative affect (*α* = 0.82), and post-stimuli disgust (*α* = 0.87).

### Results

**Figure 3:**
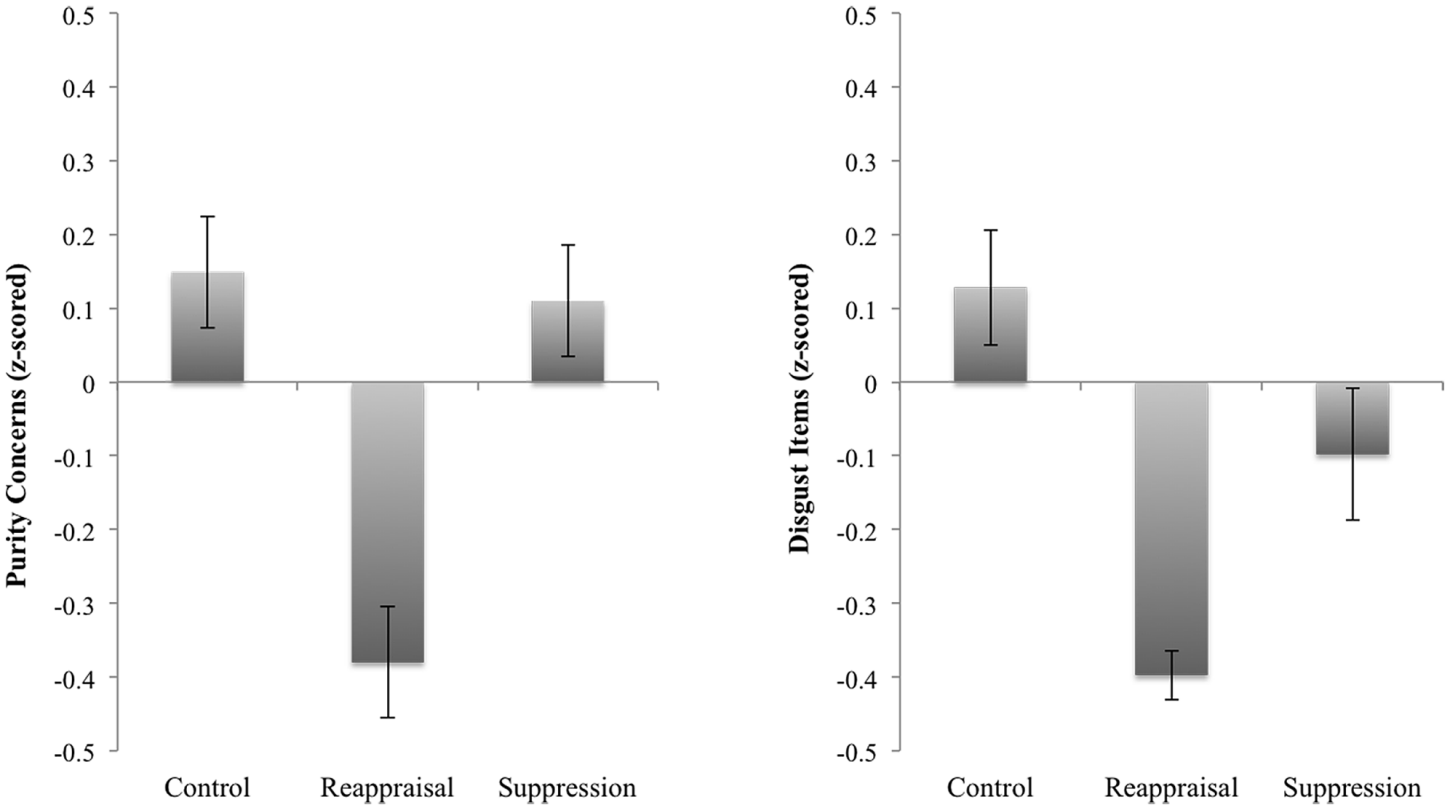
Effects of different emotion-regulation strategies used in Experiment 3. (**A**) Reappraisal significantly reduced post-stimuli disgust. (**B**) Reappraisal reduced concerns for purity as moral foundation (Error bars reflect SEM).

In the Text S1, we report the descriptive statistics of the main variables and their zero-order correlations (See Table S6 in Text S1). Experiment 2 revealed that perceived disgust from the negative stimuli differed across emotion regulation strategies, *F*(2, 109) = 3.61, *p* = 0.03 (Fig. 3). A planned contrast revealed that participants in the reappraisal condition were significantly less disgusted (*M* = –0.40, *SD* = 0.40) than were those in the control condition (*M* = 0.13, *SD* = 1.06), *p* = 0.03. Also, participants' ratings of purity as a relevant moral concern (i.e., whether one takes purity and decency into account when making judgment about right and wrong) differed significantly across the emotion regulation strategies, *F*(2, 111) = 3.59, *p* = 0.03. Participants in the reappraisal condition were less likely to perceive purity concerns as relevant to their moral judgment (*M* = –0.38, *SD* = 0.91) than were those in the control condition (*M* = 0.15, *SD* = 1.04), *p* = 0.05. Interestingly, we did not observe any statistically significant difference in other concerns for morality (harm, fairness, loyalty, and respect) across the three emotion regulation conditions, although similar patterns appeared for loyalty and respect (*p*>0.52 for all contrasts).

Lastly, we tested the hypothesis that the relationship between reappraisal and purity as a moral foundation may be explained by one's subjective feelings of disgust, and not by negative affect in general (all negative items that exclude disgust-related items). In support of this hypothesis, we found that when self-reported disgust was included as a predictor of purity concerns, the effect of reappraisal on purity concerns was no longer statistically significant (changing from B = −0.62, *SE* = 0.27, *p* = 0.02 to B = −0.43, *SE* = 0.27, *p* = 0.11). However, disgust significantly predicted purity (B = 0.27, *SE* = 0.11, *p* = 0.01). A Sobel test indicated that the reduction in regression weight was statistically significant (Z = −1.90, *p* = 0.05). A bootstrap analysis confirmed that the 95% bias-corrected confidence interval for the size of the indirect effect excluded zero (−0.38, −0.01). General feelings of negative affect, however, did not mediate the effect of reappraisal on purity concerns.

## Experiment 3

Experiment 3 tests the hypothesis that reappraisal can influence the relationship between innate disgust sensitivity and support for conservative policies. Following the same procedure as in Experiment 2, we randomly assigned participants to one of the three conditions (reappraisal, suppression, and control) and exposed them to identical disgust inducing images. However, in this study, we first measured dispositional sensitivity toward disgust before presenting the disgust images. In addition to the measures of purity concerns used in Experiment 2, we introduced an assessment of support for conservative policies.

Importantly, we also assessed the efficacy of reappraisal in regulating negative physiological arousal. Previous research using electrocardiography methods has found that disgust triggers a parasympathetic autonomic response and is characterized by a decelerated heart rate. Fear and anger, on the other hand, produce mostly a sympathetic response, and are associated with an accelerated heart rate [34], [35].

### Methods

#### Participants and Procedure

We recruited 112 individuals (*M_age_* = 35.42, *SD_age_* = 14.05; 61% male) to participate in a computer-based survey in our laboratory. During the study, participants wore non-invasive electrodes that measured heart rate. As in Experiment 2, we randomly assigned participants to one of three treatment groups (reappraisal, suppression, and no emotion regulation). The instructions and disgust inducing images remained unchanged from Experiment 2. Moreover, we expanded on Experiment 2 by analyzing the relationship between dispositional disgust sensitivity, concerns for purity, and support for conservative policies.

Before enrolling in this study, participants were required to complete an online survey containing demographic and health-related questions. This allowed us to determine whether participants were over the age of 18 and were registered to vote in the United States. We also assessed whether participants held political attitudes that fell in between liberalism and conservatism and whether they had any health conditions that could affect their physiological responses. All participants received $10 for their participation.

Once we checked physiological signals, we instructed participants to begin the study. The first task consisted of a relaxing, two-minute video. This allowed us to measure baseline physiological activities. Next, participants completed a task that assessed their sensitivity to disgust. They were then randomly assigned to one of the three conditions (reappraisal, suppression, and control) [29]. We then asked questions related to moral foundations, as well as policy preferences.

Lastly, participants completed a standard demographic questionnaire and a manipulation check. Seven participants in the reappraisal condition, and 15 participants in the suppression condition failed to use the emotion regulation strategy that they were assigned, and were thus excluded from further analysis [32]. Thus, a total of 90 participants were included in the analysis (26 reappraisers, 31 suppressors, and 33 controls).

#### Measures

We measured participants' propensity to feel disgusted (DS, *α* = 0.88) [36]. Because the DS score remains stable over time, it has been found to be a good predictor of one's behavioral willingness to engage in disgusting actions [37]. In Part I, participants rated their agreement with 14 statements (e.g., “It would bother me tremendously to touch a dead body”) on a scale from 1 (*strongly disagree*) to 5 (*strongly agree*). In Part II, participants rated 13 statements (e.g., “You are walking barefoot on concrete, and you step on an earthworm”) on their perceived disgust using a scale from 1 (*not disgusting at all*) to 5 (*extremely disgusting*).

In Experiment 3, we replaced the self-report measures of disgust with electrocardiography methods. This served to reduce self-report bias, and to track participants' emotional state closely and continuously (See Text S1). We used the 28-item MFQ (both Part I and II) [33] to assess participants' attitude toward moral principles. These measures included harm (*α* = 0.59), fairness (*α* = 0.66), loyalty (*α* = 0.63), authority (*α* = 0.63), and purity (*α* = 0.74). As before we used the 32-item measure to indicate the extent of support for conservative policies (*α* = 0.83) [27].

### Results

In the Text S1, we report the descriptive statistics of the main variables and their zero-order correlations (See Table S7 in Text S1). We first confirmed whether reappraisal mitigated the physiological effect of observing disgusting images using a mixed ANOVA, with heart rate as the dependent variable, time (baseline vs. disgust period) as the within-subjects factor, and type of emotion regulation strategy (reappraisal vs. suppression vs. no strategy employed) as the between-subjects factor. Our hypothesis was confirmed. We observed a significant decrease of heart rate over time, *F*(1, 86) = 6.27, *p* = 0.01, *η_ρ_* ^^2^^ = 0.07. Although there was no significant difference in mean heart rate across emotion regulation strategies, *F*(2, 86) = 0.48, *p* = 0.62, there was a significant interaction between the type of emotion regulation strategy used and heart rate over time, *F*(2, 86) = 3.00, *p* = 0.05, *η_ρ_* ^^2^^ = 0.06. That is, changes in heart rate depended on the emotion regulation strategy that participants had been randomly assigned. In particular, a planned contrast revealed that the heart rate for reappraisers did not decrease significantly from time  = T1 (*M* = 74.75, *SD* = 14.98) to time  = T2 (*M* = 74.33, *SD* = 15.50), *p* = 0.73, *d* = 0.03. On the other hand, heart rate decreased significantly for both suppressors and controls; for suppressors, heart rate decreased from T1 (*M* = 74.47, *SD* = 13.18) to T2 (*M* = 70.67, *SD* = 10.81), *p* = 0.02, *d* = 0.31, and for controls heart rate decreased from T1 (*M* = 73.60, *SD* = 10.63) to T2 (*M* = 70.57, *SD* = 9.72), *p* = 0.003, *d* = 0.30. This confirms the hypothesis that reappraisal not only reduces subjective feelings of disgust, but also attenuates physiological reactions to disgusting stimuli.

**Figure 4:**
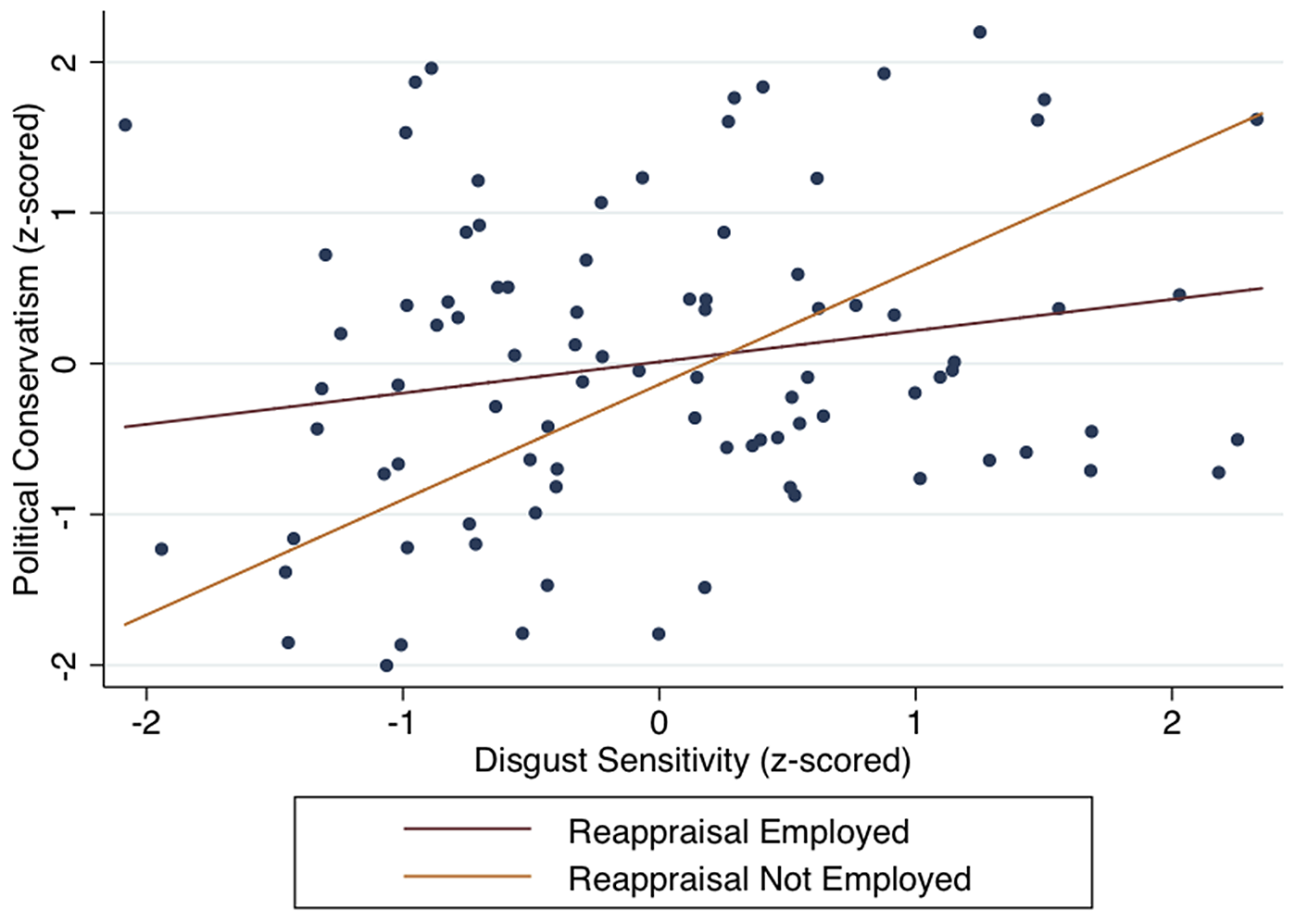
The relationship between disgust sensitivity and support for conservative policies depends on whether reappraisal is employed or not, Experiment 4. Simple slopes analysis demonstrates that when reappraisal is not used, the relationship between disgust sensitivity and support for conservative policies is statistically significant, B = 0.76, *p* = 0.004, but when reappraisal is employed, the effect is attenuated, B = 0.20, *p* = 0.55.

Next, we tested the moderation hypothesis: the relationship between innate disgust sensitivity and support for conservative policies depends on the use of reappraisal. As predicted, a significant interaction between disgust sensitivity and support for conservative policies was found (B = −0.61, *SE* = 0.26, *p* = 0.02). These tests also accounted for potential correlations with standard demographic variables (see Table S8 in Text S1). Fig. 4 shows that when reappraisal was not used, the relationship between disgust sensitivity and support for conservative policies was statistically significant, B = 0.82, *SE* = 0.26, *p* = 0.003. However, when reappraisal was employed, the effect was not significant, B = 0.25, *SE* = 0.13, *p* = 0.06. This finding suggests that reappraisal has an important role in attenuating the effect of disgust sensitivity on political attitudes.

**Figure 5:**
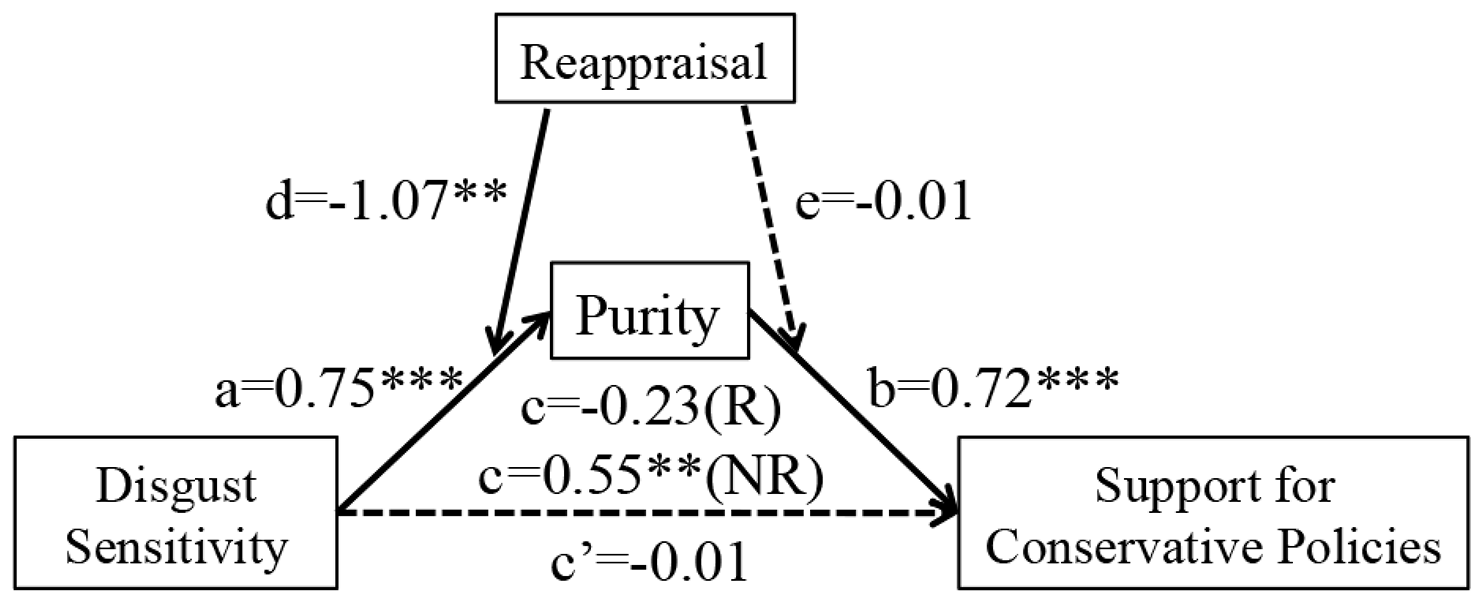
A model of moderated mediation shows that reappraisal attenuates the otherwise robust relationship between disgust sensitivity and purity concerns, thus leading to less support for conservative policies, Experiment 4. All values are regression coefficients. Purity variable consists of the items that suggest purity as a moral concern. Solid lines indicate significant paths and dashed lines indicate non-significant paths. *p<0.05, **p<0.01, ***p<0.001. c indicates the conditional indirect effect of disgust sensitivity on support for conservative policies, whereas c′ indicates direct effect. (R) and (NR) denote reappraisal and non-reappraisal conditions respectively. Binary indicator variable for suppression was entered as a covariate. All beta coefficients (a through e) are unstandardized. Standard errors, p-values, and 95% confidence intervals for the corresponding coefficient estimates are as follows. a = 0.75, *SE* = 0.64, *p*<0.001. b = 0.72, *SE* = 0.10, *p*<0.001. c = −0.23 (R), *SE* = 0.21, *p* = 0.29, (−0.65, 0.29). c = 0.55 (NR), *SE* = 0.17, p = 0.002, (0.25, 0.91). c′ = 0.01, *SE* = 0.14, *p* = 0.97, (−0.29, 0.28). d = −1.07, *SE* = 0.37, *p* = 0.005. e = −0.01, *SE* = 0.19, *p* = 0.96.

Building on the Experiment 2 result that reappraisal of disgust decreased concerns for purity as a moral foundation, we tested a related hypothesis in Experiment 3 that the interaction between disgust sensitivity and reappraisal may also predict concerns for purity, which is a potential mechanism that explains how reappraisal and disgust sensitivity are related to support for conservative policies (see Table S9 in Text S1). Fig. 5 shows the results of a complete model of moderated mediation [38], which examined whether purity concerns continue to mediate the relationship between disgust sensitivity and support for conservative policies when reappraisal was used or not used. Reappraisal significantly attenuated the association between disgust sensitivity and purity concerns (d = −1.07, *SE* = 0.37, *p* = 0.005) but had no statistically significant effect on purity concerns and support for conservative policies (e = −0.01, *SE* = 0.19, *p* = 0.96). When we accounted for purity concerns, disgust sensitivity no longer predicted support for conservative policies (c′ = 0.01, *SE* = 0.14, *p* = 0.97), but purity did (b = 0.72, *SE* = 0.10, *p*<0.001). A Sobel test indicated that the reduction in the regression weight (c→c′) was statistically significant (Z = −2.83, *p* = 0.005). Also as shown in Fig. 4, the indirect effect of disgust sensitivity on support for conservative policies was statistically significant only when reappraisal was not employed (c = −0.55, *SE* = 0.17, *p* = 0.002) but not significant when reappraisal was employed (c = −0.23, *SE* = 0.21, p = 0.29). In addition, a bootstrap analysis consisting of 1,000 samples confirmed that the 95% bias-corrected confidence intervals excluded zero in all of the significant paths presented in Fig. 5. Together, these results suggest that reappraisal may interfere with the process by which one forms moral intuitions, especially those related to purity, and thus may weaken the relationship between felt disgust and support for conservative policies. In other words, when disgust-prone individuals employ reappraisal, they may be less likely to form moral and political intuitions from their experience of disgust.

## General Discussion

In the last decade, a wide variety of research has indicated that genetic variation plays an important role in explaining the variation in political attitudes [39]–[44]. For example, attitudes toward certain political issues, such as abortion and gay rights, may be partially biologically inherited from parents [42], and fear dispositions and attachment also significantly predict political preferences toward out-group members [44].

An interesting possibility, then, is that liberals and conservatives may also exhibit biological differences in the way they deal with emotionally-arousing situations. Emerging evidence in neurophysiology has shown that being liberal is associated with having a larger anterior cingulate cortex (an area that regulates emotional processes) and stronger brain activity in this region [6], [7], [45].

Here, we show that both political ideology and support for policies are associated with the trait-based use of emotion-regulation strategies (Studies 1a and 1b). We also demonstrated that an emotion-regulation strategy can influence both emotional reactions and preference for conservative policies (Studies 2 and 3). In particular, the use of reappraisal not only reduces the psychological and physiological experience of disgust, but also buffers the effect of trait-based disgust sensitivity on purity-based moral judgments and support for conservative policies (Experiment 3). These results suggest that while political attitudes may be rooted in biologically inherited processes that influence the way we experience emotion, they are also malleable, as reappraisal is a strategy that can be learned.

An intriguing question remains whether members of a population can employ systematic efforts to promote reappraisal in order to become more politically tolerant of an out-group (such as sexual, ethnic, and religious minorities) over time. Our findings reveal a specific pattern highlighting the role of regulating disgust, suggesting that reappraisal may attenuate purity-related moral concerns and political attitude. This may in turn influence attitudes on controversial political issues such as immigration and gay marriage. In support of this finding, training individuals in reappraisal strategies in the context of the Israeli - Palestinian conflict has been found to reduce anger in conflict situations and increase preference for conciliatory solutions over aggressive policies [46], [47]. In addition, the use of reappraisal enabled individuals to reduce political intolerance of out-group members by decreasing negative emotions and increasing democratic values [48].

While previous research has focused on reappraising negative emotions towards an out-group, our study is the first to demonstrate that reappraising incidental disgust, an emotion which is unrelated to subsequent moral and political judgments, plays an important role in reducing support for conservative policies. Our method clearly isolated the effects of disgust reappraisal, reducing the influence of incidental disgust on support for conservative policies. Notably, our findings indicate that both the subjective and physiological experiences of disgust can be successfully alleviated with targeted reappraisal. This suggests that regulating incidental disgust reduces a psychologically aversive state, which in turn alters one's attitudes toward for conservative policies.

In this study, we focused on one pathway through which individuals may override the effect of emotions on political attitudes: disgust reappraisal in terms of trait sensitivity and situational experience. However, disgust may be one of many emotions that can affect the development of moral intuitions and shape support for conservative policies. Thus, we suggest that disgust reappraisal is one of many potential mechanisms behind emotion, emotion regulation, and conservatism. Our model does not suggest that a single use of reappraisal would change deep-seated ideology; instead, our data indicates that successful regulation of incidental disgust may at least temporarily change one's political disposition by reducing the tendency to form moral intuitions based on purity concerns. Future research should explore the effect of regulation on other emotions, like empathy, over which applying some degree of self-control may alter both moral judgments and political attitudes.

## Supporting Information

Text S1 **Materials and Methods.** This supplementary information includes: (i) details of the measure of support for conservative policies used in Experiment 1a and 3, and (ii) tables that represent descriptive statistics, zero-order correlations among key variables, and multivariate regression analyses.
(DOCX)
