# Supplementary material for: Emotion Regulation as the Foundation of Political Attitudes: Does Reappraisal Decrease Support for Conservative Policies?
Source: PLoS One. 2013 Dec 18;8(12):e83143. doi: 10.1371/journal.pone.0083143 (PMC3867439; doi:10.1371/journal.pone.0083143)
Supplement: Text S1 — Materials and Methods. This supplementary information includes: (i) details of the measure of support for conservative policies used in Experiment 1a and 3, and (ii) tables that represent descriptive statistics, zero-order correlations among key variables, and multivariate regression analyses. (DOCX) [file pone.0083143.s001.docx]

**Supplementary Information Text S1 for**

**Emotion Regulation as the Foundation of Political Attitudes:**

**Does Reappraisal Decrease Support for Conservative Policies?**

Jooa Julia Lee,^1^ Yunkyu Sohn,^2^ James H. Fowler^2,3^

*^1^Harvard Kennedy School, Harvard University, Cambridge, Massachusetts, United States of America*

*^2^Department of Political Science, University of California San Diego, La Jolla, California, United States of America*

*^3^Division of Medical Genetics, University of California San Diego, La Jolla, California, United States of America*

**Materials and Methods**

This supplementary information includes: (i) details of the measure of support for conservative policies used in Experiment 1a and 3, and (ii) tables that represent descriptive statistics, zero-order correlations among key variables, and multivariate regression analyses.

*Measures (Experiment 1a and Experiment 3).*

The following 32-item measure of support for conservative policies [1] was adapted and used for Experiment 1a and Experiment 3.

| Death penalty | Evolutionary theory (reverse-coded) | White superiority | Working mothers (reverse-coded) |
| --- | --- | --- | --- |
| Divine law | Pornography (reverse-coded) | Disarmament (reverse-coded) | Loyalty |
| Legal abortion (reverse-coded) | Church authority | Fluoridation (reverse-coded) | Strict rules |
| Corporal punishment | Socialism (reverse-coded) | Hippies (reverse-coded) | Racial segregation |
| Moral training | Censorship | Birth control (reverse-coded) | Foreign immigration (reverse-coded) |
| Cousin marriage (reverse-coded) | Bible truth | Military drill | Inborn conscience |
| Patriotism | School uniforms | Student pranks (reverse-coded) | Teen drivers (reverse-coded) |
| Chastity | Striptease shows (reverse-coded) | Euthanasia (reverse-coded) | Gay marriage (reverse-coded) |

*Physiological Measures (Experiment 3).*

The autonomic nervous system (ANS), which is part of the peripheral nervous system, serves a regulatory function by helping the human body adapt to internal and external demands [2]. The use of ANS measures has several benefits. First, ANS measures are unlikely to be voluntarily controlled [3]. Second, ANS measures precede conscious awareness [4].

All physiological data were scored using Mindware software modules (Mindware Technologies, Gahanna, OH) by the research assistants, who were blind to both the study hypotheses and conditions. In addition, we rescored a subsample to ensure reliability. *Heart rate* (beat per minute) was assessed through electrocardiogram recordings. We used the standard method to measure the extent to which each participant is physiologically reactive to negative stimuli, by comparing physiological responses between the baseline period (T1: while participants were watching the relaxing movie) and the disgust period (T2: while they view the disgusting images). We failed to collect heart rate data for 1 participant due to measurement issues. In addition, we statistically identified outliers as those whose heart rate was outside of 3 standard deviations from the mean during the disgust period. We thus excluded them from further analyses (2.67%; 3 out of 112 participants).

**References to Materials and Methods**

1. Wilson G, Patterson J (1968) A new measure of conservatism*. *Br J Soc Clin Psychol* 7(4):264–269.
2. Mendes WB (2009) in *Methods in Social Neuroscience,* eds Harmon-Jones, E., & Beer, J. (New York, Guilford Press), pp.118-147.
3. Gardner W, Gabriel S, Diekman A (2000) in *Handbook of psychophysiology (2nd ed)*, pp. 643-664.
4. Bechara A, Damasio H, Tranel D, Damasio A (1997) Deciding advantageously before knowing the advantageous strategy. *Science* 275(5304): 1293-1295.

**Supplementary Tables**

Table S1. A single-factor solution for the support for conservative policies

| Items | Factor loading |
| --- | --- |
| Death penalty | 0.09 |
| Divine law | -0.56 |
| Legal abortion | 0.43 |
| Corporal punishment | -0.44 |
| Moral training | 0.70 |
| Cousin marriage | -0.66 |
| Patriotism | -0.11 |
| Chastity | 0.33 |
| Evolutionary theory | -0.61 |
| Pornography | 0.70 |
| Church authority | -0.17 |
| Socialism | 0.37 |
| Censorship | 0.18 |
| Bible truth | -0.12 |
| School uniforms | -0.11 |
| Striptease shows | 0.48 |
| White superiority | 0.57 |
| Disarmament | 0.49 |
| Fluoridation | -0.41 |
| Hippies | -0.39 |
| Birth control | -0.26 |
| Military drill | 0.80 |
| Student pranks | 0.24 |
| Euthanasia | 0.31 |
| Working mothers | 0.20 |
| Loyalty | 0.45 |
| Strict rules | -0.18 |
| Racial segregation | -0.17 |
| Foreign immigration | 0.67 |
| Inborn conscience | -0.62 |
| Teen drivers | -0.41 |
| Gay marriage | -0.72 |

Note: Eigenvalue=6.64, Cronbach’s *α*=0.84.

Table S2. Zero-order correlations among key demographic variables, support for conservative policies, emotion-regulation styles, and transient mood in Experiment 1a.

|  |  | Mean | SD | (1) | (2) | (3) | (4) | (5) | (6) | (7) | (8) |
| --- | --- | --- | --- | --- | --- | --- | --- | --- | --- | --- | --- |
| (1) | Support for Conservative Policies | 0 | 1 | 1 |  |  |  |  |  |  |  |
| (2) | Reappraisal Frequency | 4.89 | 1.06 | -0.22* | 1 |  |  |  |  |  |  |
| (3) | Suppression Frequency | 3.82 | 1.33 | 0.03 | -0.07 | 1 |  |  |  |  |  |
| (4) | Positive Affect | 3.88 | 1.39 | -0.02 | 0.37*** | -0.07 | 1 |  |  |  |  |
| (5) | Negative Affect | 1.52 | 0.81 | 0.16 | -0.13 | 0.04 | 0.04 | 1 |  |  |  |
| (6) | Sex | 0.48 | 0.50 | 0.13 | 0.19* | 0.01 | 0.03 | -0.15 | 1 |  |  |
| (7) | Age | 35.21 | 12.37 | 0.20* | -0.06 | -0.14 | 0.05 | -0.19* | 0.33*** | 1 |  |
| (8) | Education | 4.04 | 1.28 | -0.02 | 0.01 | 0.15 | -0.03 | -0.07 | 0.06 | -0.05 | 1 |
| (9) | Income | 3.69 | 2.67 | -0.16 | 0.17 | -0.24* | 0.12 | 0.02 | -0.03 | 0.07 | 0.16 |

Note: **p*<0.05, ***p*<0.01, ****p*<0.001; a factor score was used for the summary variable indicating one’s support for conservative policies.

Table S3*.* OLS regression model: Support for Conservative Policies, Experiment 1a.

|  | Model (1) | Model (2) | Model (3) | Model (4) | Model (5) |
| --- | --- | --- | --- | --- | --- |
|  |  |  |  |  |  |
| Reappraisal Frequency | -0.17** |  | -0.17** | -0.19** | -0.22** |
|  | (0.06) |  | (0.06) | (0.06) | (0.07) |
| Suppression Frequency |  | 0.02 | 0.01 | 0.02 | 0.02 |
|  |  | (0.05) | (0.05) | (0.06) | (0.06) |
| Age |  |  |  | 0.01 | 0.01 |
|  |  |  |  | (0.01) | (0.01) |
| Education |  |  |  | -0.00 | 0.01 |
|  |  |  |  | (0.06) | (0.07) |
| Income |  |  |  | -0.04 | -0.04 |
|  |  |  |  | (0.03) | (0.03) |
| Sex |  |  |  | 0.20 | 0.22 |
|  |  |  |  | (0.17) | (0.17) |
| Positive Affect |  |  |  |  | 0.04 |
|  |  |  |  |  | (0.05) |
| Negative Affect |  |  |  |  | 0.20 |
|  |  |  |  |  | (0.12) |
| Constant | 0.61 | -0.33 | 0.56 | 0.07 | -0.54 |
|  | (0.33) | (0.22) | (0.38) | (0.53) | (0.55) |
| Observations | 120 | 120 | 120 | 112 | 112 |
| R-squared | 0.05 | 0.00 | 0.05 | 0.13 | 0.17 |
| Adjusted R-squared | 0.04 | -0.00 | 0.03 | 0.08 | 0.10 |

Note: Standard errors in parentheses; **p*<0.05, ***p*<0.01, ****p*<0.001

Table S4*.* Zero-order correlations among key demographic variables, political orientation, emotion-regulation styles, and transient mood in Experiment 1b.

|  |  | Mean | SD | (1) | (2) | (3) | (4) | (5) | (6) | (7) | (8) |
| --- | --- | --- | --- | --- | --- | --- | --- | --- | --- | --- | --- |
| (1) | Political Conservatism | 2.96 | 1.39 | 1 |  |  |  |  |  |  |  |
| (2) | Reappraisal Frequency | 4.93 | 1.01 | -0.16* | 1 |  |  |  |  |  |  |
| (3) | Suppression Frequency | 3.71 | 1.15 | 0.15* | -0.01 | 1 |  |  |  |  |  |
| (4) | Positive Affect | 2.29 | 0.87 | -0.00 | 0.08 | 0.04 | 1 |  |  |  |  |
| (5) | Negative Affect | 1.62 | 0.52 | 0.09 | -0.04 | 0.13 | 0.17* | 1 |  |  |  |
| (6) | Sex | 0.46 | 0.50 | -0.08 | 0.23** | -0.20** | 0.19** | 0.04 | 1 |  |  |
| (7) | Age | 25.21 | 4.01 | 0.05 | -0.01 | 0.20** | 0.20** | 0.20** | 0.04 | 1 |  |
| (8) | Education | 2.63 | 1.23 | -0.05 | 0.02 | 0.06 | 0.06 | 0.21** | 0.09 | 0.65*** | 1 |
| (9) | Income | 6.09 | 3.81 | 0.05 | 0.12 | 0.13 | 0.13 | -0.03 | -0.02 | 0.07 | 0.08 |

Note: **p*<0.05, ***p*<0.01, ****p*<0.001

Table S5*.* OLS regression model: Self-reported Political Conservatism, Experiment 1b.

|  | Model (1) | Model (2) | Model (3) | Model (4) |
| --- | --- | --- | --- | --- |
|  |  |  |  |  |
| Reappraisal Frequency | -0.22* | -0.22* | -0.23* | -0.22* |
|  | (0.11) | (0.11) | (0.11) | (0.11) |
| Suppression Frequency |  | 0.17 | 0.16 | 0.14 |
|  |  | (0.09) | (0.09) | (0.09) |
| Age |  |  | 0.04 | 0.04 |
|  |  |  | (0.03) | (0.03) |
| Education |  |  | -0.15^ | -0.17^ |
|  |  |  | (0.09) | (0.09) |
| Income |  |  | 0.03 | 0.03 |
|  |  |  | (0.03) | (0.03) |
| Sex |  |  | -0.03 | -0.07 |
|  |  |  | (0.20) | (0.20) |
| Positive Affect |  |  |  | -0.07 |
|  |  |  |  | (0.11) |
| Negative Affect |  |  |  | 0.22 |
|  |  |  |  | (0.20) |
| Constant | 4.06*** | 3.41*** | 2.77*** | 2.61*** |
|  | (0.57) | (0.64) | (1.14) | (1.17) |
| Observations | 199 | 199 | 199 | 199 |
| R-squared | 0.03 | 0.05 | 0.07 | 0.07 |
| Adjusted R-squared | 0.02 | 0.04 | 0.03 | 0.03 |

Note: Standard errors in parentheses; **p*<0.05, ***p*<0.01, ****p*<0.001

Table S6. Zero-order correlations among indicators of reappraisal and suppression, emotions, and concerns for purity as moral foundation in Experiment 2.

|  |  | Mean | SD | (1) | (2) | (3) | (4) |
| --- | --- | --- | --- | --- | --- | --- | --- |
| (1) | Purity concerns | 0 | 1 | 1 |  |  |  |
| (2) | Post-stimuli negative affect | 0 | 1 | 0.19* | 1 |  |  |
| (3) | Post-stimuli disgust items | 0 | 1 | 0.27** | 0.89*** | 1 |  |
| (4) | Reappraisal dummy | 0.29 | 0.45 | -0.23** | -0.23** | -0.23** | 1 |
| (5) | Suppression dummy | 0.23 | 0.42 | 0.06 | -0.04 | -0.05 | -0.35*** |
|  |  |  |  |  |  |  |  |

Note: **p*<0.05, ***p*<0.01, ****p*<0.001

Table S7. Zero-order correlations among dispositional disgust sensitivity, indicators of reappraisal and suppression, purity concerns as moral foundation, and support for conservative policies in Experiment 3.

|  |  | Mean | SD | (1) | (2) | (3) | (4) | |
| --- | --- | --- | --- | --- | --- | --- | --- | --- |
| (1) | Support for conservative policies | 0 | 1 | 1 |  |  |  | |
| (2) | Purity concerns | 0 | 1 | 0.68*** | 1 |  |  | |
| (3) | Disgust sensitivity | 0 | 1 | 0.16 | 0.25* | 1 |  | |
| (4) | Reappraisal dummy | 0.29 | 0.46 | 0.08 | -0.02 | -0.13 | 1 | |
| (5) | Suppression dummy | 0.34 | 0.48 | -0.05 | 0.18 | 0.07 | -0.46*** | |
|  |  |  |  |  |  |  |  |  |

Note: **p*<0.05, ***p*<0.01, ****p*<0.001; factor scores were used for all three summary variables.

Table S8. OLS regression model: Support for conservative policies, Experiment 3.

|  | Model (1) | Model (2) |
| --- | --- | --- |
| Reappraisal dummy | 0.16 | 0.15 |
|  | (0.26) | (0.27) |
| Suppression dummy | -0.02 | -0.00 |
|  | (0.24) | (0.25) |
| Disgust sensitivity | 0.49** | 0.54** |
|  | (0.17) | (0.17) |
| Interaction term | -0.61* | -0.62* |
| (DS x Reappraisal) | (0.25) | (0.26) |
| Interaction term | -0.39 | -0.44 |
| (DS x Suppression) | (0.25) | (0.26) |
| Age |  | 0.00 |
|  |  | (0.01) |
| Sex (1=male, 2=female) |  | -0.21 |
|  |  | (0.25) |
| Income |  | 0.09 |
|  |  | (0.06) |
| Education |  | -0.00 |
|  |  | (0.08) |
| Constant | -0.06 | -0.19 |
|  | (0.17) | (0.54) |
| Observations | 90 | 90 |
| R-squared | 0.10 | 0.13 |
| Adjusted R-squared | 0.05 | 0.03 |
| RMSE | 0.97 | 0.98 |

Note: Standard errors in parentheses, **p*<0.05, ***p*<0.01, ****p*<0.001.

Table S9*.* OLS regression models: Five moral foundations, Experiment 3.

| Dependent Measures | Harm | Fairness | Royalty | Respect | Purity |
| --- | --- | --- | --- | --- | --- |
|  |  |  |  |  |  |
| Reappraisal dummy | 0.25 | -0.13 | 0.10 | 0.66 | 0.36 |
|  | (0.40) | (0.38) | (0.45) | (0.39) | (0.47) |
|  |  |  |  |  |  |
| Suppression dummy | 0.31 | -0.00 | -0.38 | -0.16 | 0.99* |
|  | (0.37) | (0.36) | (0.43) | (0.36) | (0.45) |
|  |  |  |  |  |  |
| Disgust sensitivity | 0.25 | -0.05 | 0.32 | 0.81** | 1.21*** |
|  | (0.26) | (0.25) | (0.29) | (0.25) | (0.31) |
|  |  |  |  |  |  |
| Interaction term | 0.18 | 0.22 | -0.20 | -0.17 | -1.43** |
| (DS * Reappraisal) | (0.39) | (0.38) | (0.44) | (0.38) | (0.46) |
|  |  |  |  |  |  |
| Interaction term | -0.23 | 0.30 | 0.22 | -0.35 | -0.57 |
| (DS * Suppression) | (0.38) | (0.37) | (0.44) | (0.37) | (0.46) |
|  |  |  |  |  |  |
| Constant | 8.64*** | 9.26*** | 7.08*** | 7.16*** | 5.74*** |
|  | (0.26) | (0.25) | (0.30) | (0.25) | (0.31) |
|  |  |  |  |  |  |
| Observations | 90 | 90 | 90 | 90 | 90 |
| R-squared | 0.04 | 0.02 | 0.06 | 0.20 | 0.22 |
| Adjusted R-squared | -0.01 | -0.04 | -0.00 | 0.15 | 0.18 |
| RMSE | 1.49 | 1.45 | 1.70 | 1.45 | 1.78 |

Note: Standard errors in parentheses, **p*<0.05, ***p*<0.01, ****p*<0.001.
